# Thrombus in Transit through Patent Foramen Ovale

**DOI:** 10.1155/2013/395879

**Published:** 2013-09-30

**Authors:** Hassan Baydoun, Iskander Barakat, Elie Hatem, Michel Chalhoub, Ali Mroueh

**Affiliations:** ^1^Department of Medicine, Staten Island University Hospital, NY 10305, USA; ^2^Department of Pulmonary and Critical Care, Staten Island University Hospital, NY 10305, USA; ^3^Department of Cardiology, Faculty of Medical Sciences, Lebanese University, Lebanon

## Abstract

A thrombus in transit through a patent foramen ovale (PFO) with impending paradoxical embolism is an extremely rare event. Due to its transient nature, it is unable to identify the thrombus, and most of the cases have been reported at autopsy. We are reporting a case of thrombus straddling the foramen ovale which was diagnosed by echocardiography and treated surgically. Through this personal case, an exhaustive review of the literature was performed. There were 88 cases reported. We concluded that there is no medical consensus about the best option for treatment. Nevertheless, surgery, which is associated with fewer complications of recurrent embolic events than those of thrombolysis and anticoagulation, appeared to be the best approach in patients who are not at a high surgical risk. Anticoagulant treatment appears to be an acceptable therapeutic alternative to surgery, particularly in patients with comorbidities who are at high surgical risk and for patients with small PFO. Thrombolysis is linked to the highest mortality, which could be explained by the severity of the patient's initial presentation. In conclusion, and after the cumulative effects of these case reports, we propose a diagram consisting of the use of the three therapeutic options in the different clinical scenarios.

## 1. Introduction

Patent foramen ovale (PFO) is an increasingly investigated cause of cryptogenic embolic stroke. An entrapped embolus through this foramen is an extremely rare finding. The first reported case diagnosed by echocardiography was by Nellessen et al. in 1985 [1] with less than hundred cases being reported so far in the literature. Due to its transient nature, it is virtually impossible to identify the embolus at the time of clinical presentation, and most of the cases have been diagnosed at autopsy. However, with the advance in imaging technology, the identification of this condition has become quicker, but the management remains uncertain due to its rarity. Therapeutic options include medical treatment with heparin or thrombolysis and surgical thrombectomy. We are reporting a case of thrombus in transit through PFO with impending paradoxical embolism which was diagnosed by echocardiography and treated surgically. We are also proposing, after reviewing all the literature, a diagram for the three therapeutic options available in the different clinical scenarios.

## 2. Case Presentation

A 48-year-old man male presented to the emergency department (ED) with a two-day history of progressive shortness of breath. He was seen earlier that day at another facility for the same reason and was diagnosed with decompensated heart failure and discharged home on oral furosemide. The patient denied any chest pain or palpitations but reported to have orthopnea, dyspnea at rest, nausea, and dizziness. Review of system was negative.

On physical examination, the patient appeared in mild distress. He was found to have tachycardia with a heart rate of 105 beats per minute and tachypnea with a respiratory rate of 24 per minute and had a high blood pressure of 175/76 mm Hg. His oxygen saturation was 95% on 3 liters of oxygen per min through a nasal cannula. Mild jugular veins distention was noted. Examination of the heart showed regular heart sounds with 2/6 systolic murmur at right sternal border. Fine basal rales were heard throughout both lungs. Moderate (2+) bilateral lower extremity pitting edema was noticed. The rest of the physical examination was normal.

His past medical history is significant for heart failure with preserved ejection fraction, hypertension, diabetes mellitus type II, chronic kidney disease stage III, obstructive sleep apnea, and schizophrenia.

His medications included insulin, furosemide, lisinopril, metoprolol, aspirin, and atorvastatin; he states that he is not very compliant with his medications. 

His blood work revealed two sets of cardiac enzymes within normal limits (troponin of 0.2 and 0.18 ng/mL, resp.). His brain natriuretic peptide (BNP) was elevated (365 pg/mL). EKG showed sinus tachycardia with nonspecific ST-T changes. Chest X-ray revealed mild interstitial edema and cardiomegaly. The patient was admitted with a presumed diagnosis of decompensated heart failure. He was started on intravenous diuretics and sent to the telemetry unit. The transthoracic echocardiogram (TTE) showed normal left ventricular systolic function, moderate concentric hypertrophy, and paradoxical motion of the ventricular septum. The right atrium (RA) and ventricle (RV) were dilated; this was associated with RV strain and moderately elevated pulmonary systolic pressure. A large serpiginous thrombus in the RA extending to the RV across the tricuspid valve was seen. The patient was started on intravenous heparin drip. A bedside bilateral lower extremities duplex showed right tibial and popliteal veins thrombosis. 

**Figure 1:**
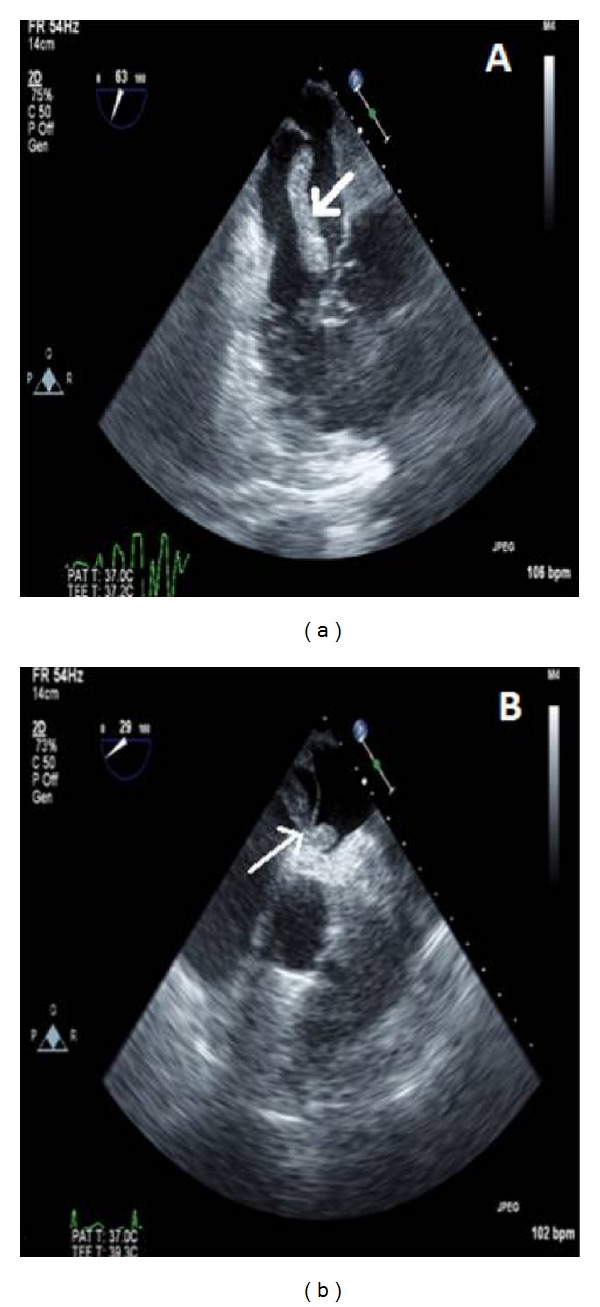
Transesophageal echocardiogram images showing a large snake-like thrombus in the RA (a) trapped in a PFO and extending into the left atrium (b).

A multidisciplinary team consisting of a cardiologist, cardiothoracic surgeon, and a pulmonologist decided to take the patient emergently to the operating room for cardiac thrombectomy and possible pulmonary embolectomy. A preoperative transesophageal echocardiogram (TEE) was performed to confirm the diagnosis. It showed a large firm cylindrical thrombus floating in the RA with a segment being entrapped through a PFO (Figure 1). Cardiac thrombectomy with careful extraction of the thrombus was performed in addition to the closure of the PFO. The patient was transferred to the cardiothoracic unit. He remained intubated for a total of 6 days after which he was extubated and transferred to the floor. He was started on long-term anticoagulation (warfarin) and discharged safely after 14 days of hospitalization. 

## 3. Discussion

A thrombus straddling a patent foramen ovale (PFO) is a very rare event. The most common presentation is paradoxical embolism which requires the passage of an embolus from the venous to the arterial system, and in this case, through a PFO. The incidence of PFO is around 27% through all ages [1–3]. This passage needs a higher right than left atrial pressure, which happens in the context of pulmonary embolism (PE) or pulmonary hypertension [4]. 

Fauveaua et al. [5] reviewed all the reported cases (around 88 cases) published between 1985 and 2007, when the first thrombus straddling the PFO was reported. According to their review, the clinical presentation of most of the patients was not specific. The most reported symptoms were shortness of breath, chest pain, palpitations, syncope, low blood pressure, atrial fibrillation, hemoptysis, and cyanosis. Almost half of the patients were diagnosed, and they presented with an association between PE and systemic embolism before treatment. Two cases of paradoxical embolism occurred after treatment. The most frequent site of systemic embolism was cerebral; other sites of systemic embolism were the coronary arteries, kidneys, the spleen, and the inferior or superior limbs. All patients were anticoagulated. 

Thrombolysis was administered in 18% of patients. Fifty-five patients finally underwent emergency cardiotomy and thrombectomy associated with PFO closure. Patients treated with heparin and thrombolysis were older than those treated with surgery. The group treated with heparin had more frequent strokes. Most authors chose surgical treatment as the first option. Ten authors preferred the anticoagulation treatment because of important associated comorbidities including hemodynamic instability, stroke, advanced age, and progressive cancer [5]. The reasons of authors for using thrombolysis were the presence of unstable hemodynamics and comorbidities. 

Our PubMed search of the literature published till 2013 was conducted using the search terms “impending paradoxical embolism” or “thrombus in transit through PFO.” However, here, we report the cases transferred to the floor between 2008 and 2013 since the previous cases were reported in the review article described above. Ten articles were found. Nine of them were published in case-report formats. One article is a review of the literature [2]. Two cases were reported as images [6]. All patients were anticoagulated with unfractionated heparin (UH) and then discharged on long-term anticoagulation (warfarin). Thrombolysis was administered in four cases. One case was supposed to go for surgery, but the thrombi disappeared after spontaneous cough during TEE, and, due to a very high risk of paradoxical systemic embolism with potential disastrous consequences, he underwent emergent intravenous thrombolysis [7]. Five cases underwent emergent surgery which included cardiac thrombectomy and closure of the PFO.

Based on a previous review [5], anticoagulant treatment appears to be an acceptable therapeutic alternative to surgery, particularly in patients with comorbidities (e.g., increased age, stroke, and progressive cancer) who are at high surgical risk and for patients with small PFO. Surgical treatment appears justified in the prevention of paradoxical embolism and must be done without delay if it is the preferred treatment strategy. However, there was a very slight difference in mortality between patients treated with heparin and the surgical group (14% and 12%, resp.). Thrombolysis is linked to the highest mortality (36%), which could be explained by the severity of the patient's initial presentation [5].

Myers et al. [2] reviewed in 2010 the observational studies on this subject to identify prognostic factors and to compare mortality and systemic embolism between treatments. Surgical thromboembolectomy showed a nonsignificant trend toward improved survival (odds ratio (OR), 0.65 (0.24–1.72); *P* = 0.65) and significantly reduced systemic embolism and composite of mortality and systemic embolism, compared with anticoagulation alone. Thrombolysis, on the other hand, had the opposite effect, although not to a significant level (OR, 1.62 (0.43–5.97); *P* = 0.47).

**Figure 2:**
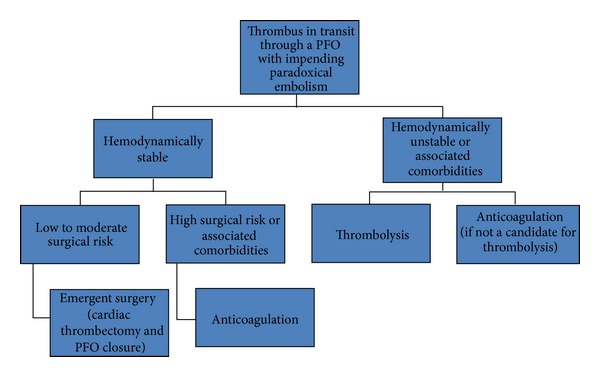
Diagram showing the different therapeutic options for a thrombus in transit through a PFO with impending paradoxical embolism.

After reviewing almost all the published cases regarding this topic, we propose the following management diagram for patients with entrapped thrombus in a patent foramen ovale which consists of three treatment regimens (Figure 2).

In conclusion, based on the cumulative results of these case reports, there is no medical consensus about the best option for treatment. Nevertheless, surgery, which is associated with fewer complications of recurrent embolic events than those of thrombolysis and anticoagulation [8], appeared to be the best approach in patients who are surgical candidates.

## References

[1] Nellessen U, Daniel WG, Matheis G (1985). Impending paradoxical embolism from atrial thrombus: correct diagnosis by transesophageal echocardiography and prevention by surgery. *Journal of the American College of Cardiology*.

[2] Myers PO, Bounameaux H, Panos A, Lerch R, Kalangos A (2010). Impending paradoxical embolism: systematic review of prognostic factors and treatment. *Chest*.

[3] Wahl A, Krumsdorf U, Meier B (2005). Transcatheter treatment of atrial septal aneurysm associated with patent foramen ovale for prevention of recurrent paradoxical embolism in high-risk patients. *Journal of the American College of Cardiology*.

[4] Cakir C, Duygu H, Eren NK, Akyildiz ZI, Nazli C, Ergene O (2008). Witnessing a rare event- Thrombus seeking its route in the right atrium: “Thrombus-in-transit”. *Journal of Cardiovascular Medicine*.

[5] Fauveaua E, Cohen A, Bonnet N, Gacem K, Lardoux H (2008). Surgical or medical treatment for thrombus straddling the patent foramen ovale: impending paradoxical embolism? Report of four clinical cases and literature review. *Archives of Cardiovascular Diseases*.

[6] Abdelsalam M, Mumtaz M, Sackman I (2012). Thrombus in transit through a patent foramen ovale. *Heart*.

[7] Kim JH, Kim YJ (2011). Thrombus in transit within a patent foramen ovale: gone with the cough!. *Journal of Cardiovascular Ultrasound*.

[8] Aboyans V, Lacroix P, Ostyn E, Cornu E, Laskar M (1998). Diagnosis and management of entrapped embolus through a patent foramen ovale. *European Journal of Cardio-thoracic Surgery*.

